# Gendered Income Trajectories and Health Disparities at Midlife: Evidence From the NLSY79 Cohort

**DOI:** 10.1093/geroni/igaf122.3778

**Published:** 2025-12-31

**Authors:** Mingyue Ma

**Affiliations:** Purdue University, West Lafayette, Indiana, United States

## Abstract

The body of literature on significant gender inequality in health in the United States, while extensive, has paid relatively little attention to how these disparities originate from longitudinal income trajectories. This study adopts a gender perspective to determine whether women’s health disadvantages stem from early experiences in the labor market. Using data from National Longitudinal Survey of Youth 1979 (NLSY79), this study applies Group-Based Trajectory Model (GBTM) to depict distinct types of time-varying income trajectories since their entry into the labor market at ages 25 until their exit at ages 49. I first use Multinomial Logistic regression to analyze gender differences in these trajectories. Further, I use Ordinary Least Square regression to predict later-life health outcomes, including physical and mental health, to investigate how income trajectories contribute to the observed disadvantage of women’s health outcomes. Finally, I employ KHB (Karlson–Holm–Breen) method to investigate whether and to what extent the mediators -income trajectories could explain the gender difference in health outcomes. I found that from a temporal perspective, women’s income trajectories are more likely to remain in low-status or show a downward trend, while men are vice versa. Compared to men, women are more likely to exhibit worse health outcomes at midlife. Further, gender differences in income trajectories significantly mediate gender inequality in physical health, while partially account for gender inequality in mental health. This evidence highlights the need for policies that secure women’s labor rights and equitable pay, as traditional female roles may impose hidden burdens that harm midlife health.

